# Realization of a CORDIC-Based Plug-In Accelerometer Module for PSG System in Head Position Monitoring for OSAS Patients

**DOI:** 10.1155/2017/4728187

**Published:** 2017-08-20

**Authors:** Wen-Yen Lin, Wen-Cheng Chou, Tsu-Hui Shiao, Guang-Ming Shiao, Chin-Shan Luo, Ming-Yih Lee

**Affiliations:** ^1^Department of Electrical Engineering, Center for Biomedical Engineering, Chang Gung University, Taoyuan, Taiwan; ^2^Division of Cardiology, Department of Internal Medicine, Chang Gung Memorial Hospital, Linkou, Taoyuan, Taiwan; ^3^Department of Electrical Engineering, Chang Gung University, Taoyuan, Taiwan; ^4^Department of Chest Medicine, Taipei Veterans General Hospital, Taipei, Taiwan; ^5^Division of Pulmonary Medicine, Department of Internal Medicine, Shuang Ho Hospital, Taipei Medical University, New Taipei City, Taiwan; ^6^Graduate Institute of Medical Mechatronics, Center for Biomedical Engineering, Chang Gung University, Taoyuan, Taiwan

## Abstract

Overnight polysomnography (PSG) is currently the standard diagnostic procedure for obstructive sleep apnea (OSA). It has been known that monitoring of head position in sleep is crucial not only for the diagnosis (positional sleep apnea) but also for the management of OSA (positional therapy). However, there are no sensor systems available clinically to hook up with PSG for accurate head position monitoring. In this paper, an accelerometer-based sensing system for accurate head position monitoring is developed and realized. The core CORDIC- (COordinate Rotation DIgital Computer-) based tilting sensing algorithm is realized in the system to quickly and accurately convert accelerometer raw data into the desired head position tilting angles. The system can hook up with PSG devices for diagnosis to have head position information integrated with other PSG-monitored signals. It has been applied in an IRB test in Taipei Veterans General Hospital and has been proved that it can meet the medical needs of accurate head position monitoring for PSG diagnosis.

## 1. Introduction

Obstructive sleep apnea syndrome (OSAS) is common in our community and is characterized by repetitive, short-duration blockages of the upper airways during sleep, resulting in episodic cessation of breathing (apnea) or reduction in airflow (hypopnea) that may lead to frequent arousals, disrupted sleep structures, excessive daytime sleepiness, intermittent hypoxemia, and many other systemic effects. Sleep apnea is estimated to occur in up to 24% of middle-aged men and 9% of middle-aged women [1]. Fortunately, clinical studies reported that the remission from apnea syndrome was diagnosed from the patients with mild OSAS. That finding can relieve the problem of sleep apnea. The reason of the remission might be caused by the change of body or head position. Studies showed that lying on one's side or the lateral decubitus position reduces the number of apneic events as compared to lying on one's back or supine sleeping position [2, 3]. Sleeping positions were suggested to have correlation with the sleeping problems. However, there is no scientific evidence to support this clinical observation.

Currently, overnight polysomnography (PSG) is the standard diagnostic procedure for OSAS. Some pilot studies showed that monitoring of head position is crucial [4, 5] not only for the diagnosis (positional sleep apnea) but also for the management of OSAS (positional therapy). However, the existing PSG is incapable of measuring head position with high accuracy for the study of remission. In fact, the researchers had to use the existing body position sensor of PSG, which usually is mounted on the trunk, and had the sensor mounted on the head for the detection of head position in order to find the correlation between head position and the remission of the OSAS. The body position sensor only identifies the position into 8 segments for 0°, −45°, −90°, −135°, −180°, −225°, −270°, and −315° ranges. Recently, some studies proposed new techniques to monitor sleeping body position. By using load cells placed under the supports of a bed, the individual's lying position was classified for the detection of sleep apnea [6, 7]. Moreover, a textile-based ECG system was developed for the determination of the sleeping body position [8]. Eight electrodes were multiplexed in the system and supported by a foam pad to ensure good contact as well as comfort. By measuring and analyzing the ECG signals, both the heart rate and sleeping body position could be determined. The aforementioned techniques only measured the sleeping body position but not the head position. The correlation between the head position and remission had not yet been investigated scientifically due to the lack of accurate head position monitoring tools for the study. Indeed, the researchers require a system which is capable of measuring the head position (orientation) with 1°~2° accuracy to hook up with PSG for further studies on the correlation between head position and the remission of OSAS patients.

In this work, a novel head position monitoring system was developed for the study of the correlation between head position and remission of OSAS. The system had the advantages of low power, low system cost, light in weight, and real-time monitoring and fulfilled the clinical requirement that eliminates the disturbance of the subjects. It can be integrated with current PSG to perform real-time diagnosis with accurate head and body position information provided. In this system, 3-axis digital accelerometers were used for the measurement of head position. However, the calculation of head titling angle from the mathematical formulas is time-consuming and cannot be applied to in-line and real-time applications. In order to shorten the time cost of calculating the angles, a novel CORDIC- (COordinate Rotation DIgital Computer-) based low-complexity and less-memory tilting sensing algorithm was used [9]. As a matter of fact, the computation time of the CORDIC-based algorithm was almost only 1/4 of the calculations from the mathematical formulas on the microcontroller used. Therefore, the embedded system could finish all tasks within every sampling period including the collection of raw data from the accelerometers, calculation of head position, and transmission of data from the microcontroller to the PSG. Hence, the system successfully performed head position monitoring in an in-line and real-time manner. Finally, a practical clinical demonstration was conducted to show the realization of using the system for the study of the correlation between head position and remission of OSAS.

## 2. Materials and Methods

### 2.1. Head Position Monitoring for OSAS Patients

**Figure 1:**
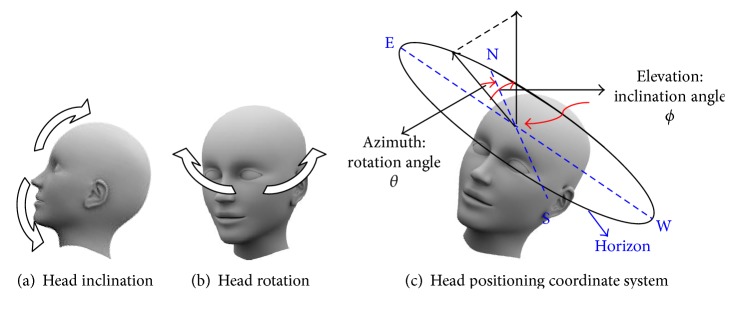
Head movements: (a) inclination, (b) rotation, and (c) head positioning coordinate system.

The typical movements of the head include inclinations (nodding), and the rotations are shown in Figures 1(a) and 1(b). In a traditional spherical coordinate system, the azimuth (rotation angle) of the object is defined as the angle between the direction of the projected object on the horizon and the north. The elevation (inclination angle) defines the angle between the direction of the object and the zenith. Intuitively, the head position can be represented by its rotation angle and inclination angle when we apply the direction of the forehead in the spherical coordinate system of the earth, as shown in Figure 1(c). In this system, accelerometers were used for the measurement of head position. Inclination or tilt angle sensing can be determined by the gravity vector and its projection on the axes. Depending on the number of axes available in the accelerometer, different inclination or tilt angles can be defined and calculated.

### 2.2. Accelerometer-Based Tilting Sensing

**Figure 2:**
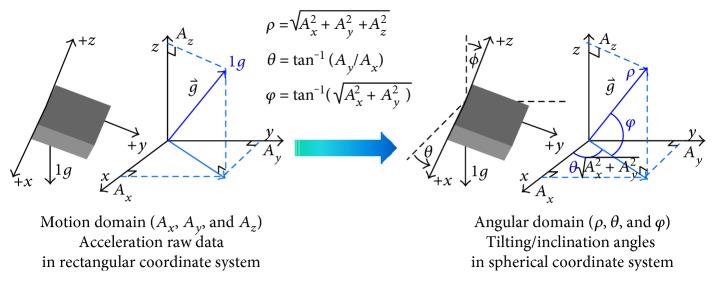
Angles of the spherical coordinate system and domain transformation.

When a 3-axis accelerometer is used for inclination sensing, the raw data read from accelerometer are the projections of the gravity vector on the three axes, *x*, *y*, and *z* (*A*_*x*_, *A*_*y*_, and  *A*_*z*_), in the rectangular coordinate system of the motion domain. To understand the orientation of the accelerometer, we can represent the gravity vector in the spherical coordinate system, (*ρ*, *θ*, and  *φ*), where *ρ* is the length of the gravity vector, that is, 1*g*, *θ* is the angle between the *x*-axis and the projected gravity vector on the *x*-*y* plane, where *φ* is the angle between the gravity vector and *x*-*y* plane. As a matter of fact, to retrieve the orientation information using the accelerometer is to perform domain transformation from the rectangular coordinate system in the motion domain of the measured raw data from the accelerometer to the spherical coordinate system in the angular domain as shown in Figure 2. The transformation equations between these two coordinate systems can be expressed in

(1) $$\begin{array}{c} A_{x}=\rho \;\cos\varphi \cdot \cos\theta ,\\ A_{y}=\rho \;\cos\varphi \cdot \sin\theta ,\\ A_{z}=\rho \;\sin\varphi , \end{array}$$

where −*π*/2 ≤ *φ* ≤ *π*/2 and –*π* ≤ *θ* ≤ *π*. So, given a reading from 3D accelerometer data (*A*_*x*_, *A*_*y*_, and  *A*_*z*_), we can calculate the two angles *φ* and *θ* as

(2) $$\begin{array}{c} \theta =\text{arctan}\left(\frac{A_{y}}{A_{x}}\right)+\sigma \cdot \lambda \cdot \pi ,\\ \varphi =\text{arctan}\left(\frac{A_{z}}{\sqrt{{A_{x}}^{2}+{A_{y}}^{2}}}\right). \end{array}$$

The term of *σ*  ·  *λ*  ·  *π* added in *θ* is to have it converted into –*π* ≤ *θ* ≤ *π* range since the original arctan function only produces –*π*/2 ≤ *θ* ≤ *π*/2 range, where *σ* = 0, if *A*_*x*_ ≥ 0; otherwise, *σ* = 1. *λ* = 1, if *A*_*y*_ ≥ 0; otherwise, *λ* = −1.

### 2.3. CORDIC-Based Tilting Sensing Algorithm

The calculations of these trigonometric functions are usually complicated; therefore, long computation time is required by low-cost and low-power microcontroller. Hence, most research works adopted offline computations [10], streaming the raw data to PC/PDA for computation [11–13], and table look up [14]. However, the offline computation approach could not identify the tilting information in real time and in the system. Even if the streaming approach could retrieve the orientation information in real time, it could not perform the calculations in the system. Therefore, these two approaches were not able to produce the in-line and real-time orientation information and could not be integrated into existing PSGs for real-time diagnosis. Even worse, the streaming approach may raise concerns of excessive RF signal transmission. Table look up approach required huge memory space for table storage, and higher system cost was induced. Huge memory space is not feasible on typical microcontrollers which usually only have few hundred Kbytes or even less of flash or ROM on chip. Based on the existing technologies, the system is not capable of performing early detection, delivering early warning and, hence, is not capable of taking early actions. Therefore, an intelligent and low-complexity CORDIC- (COordinate Rotation DIgital Computer-) based tilting angle identification algorithm based on the 2D CORDIC [15] operations is proposed for saving the computation power. This can perform the computation in (2) with only basic adders and binary shifters for rotation of a 2D vector through a sequence of simple elementary rotations.

**Figure 3:**
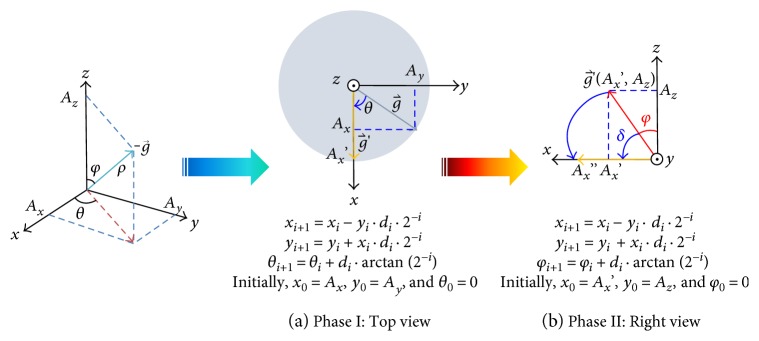
The computation process of CORDIC-based tilt sensing algorithm.

The algorithm is to calculate the angle between a 3D vector and one of the three axes. Taking the identification of the angle *φ*, for example, Figure 3 illustrates the process of the algorithm. The algorithm is conducted in two phases. Phase I, Figure 3(a), is to rotate the $-\overset{⃑}{g}$ projected vector on the *x*-*y* plane and have it aligned to the *x*-axis and forms $\overset{⃑}{g^{\prime }}$ vector as looking from the pointing direction of the *z*-axis (top view). The rotation is performed by 2D CORDIC on the *x*-*y* plane with the initial 2D vector (*A*_*x*_, *A*_*y*_) and the final vector (*A*_*x*_′, 0). The iterative equations are shown in Figure 3(a), and the rotated angle *θ* and ${A_{x}}^{\prime }=\sqrt{A_{x}^{2}+A_{y}^{2}}$ are accrued. Phase II, as shown in Figure 3(b), is to rotate the $\overset{⃑}{g^{\prime }}$ vector on the *x*-*z* plane and have it aligned to the *x*-axis, as looking from the pointing direction of the *y*-axis (right side). The rotation is performed by 2D CORDIC on the *x*-*z* plane with the initial 2D vector (*A*_*x*_′, *A*_*z*_) and have it aligned to the *x*-axis. The iterative equations are shown in Figure 3(b), and the rotated angle *δ* is accrued through CORDIC operations and *φ* = *π*/2 − *δ*. As you can see, the tilt angle, *φ*, is obtained with simple shifting and addition operations only by this proposed algorithm.

### 2.4. System Design and Realization

#### 2.4.1. Definition of Head Position Angles

**Figure 4:**
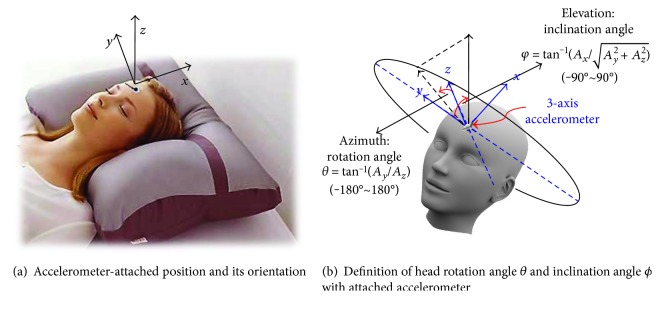
(a) Attached accelerometer on the forehead and the orientation. (b) Definition of the head position angles accordingly.

In the proposed sensing system, an accelerometer is attached to the forehead of the subject mounted with 3M™ Micropore™ Surgical Tape in the orientation shown in Figure 4(a), where *x*-axis points toward the head of the head, *y*-axis points to the right-hand side of the body, and *z*-axis points to the front of the head. With the accelerometer attached to the forehead to comply with the defined orientation, the head would rotate along the *x*-axis. Therefore, the rotation angle *θ* and the inclination angle *φ* can be calculated as in (3) for a different orientation compared with those in (2). It is also represented in Figure 4(b).

(3) $$\begin{array}{c} \varphi =\arctan\left(\frac{A_{x}}{\sqrt{{A_{y}}^{2}+{A_{z}}^{2}}}\right),\\ \theta =\arctan\left(\frac{A_{y}}{A_{z}}\right)+\sigma \cdot \lambda \cdot \pi , \end{array}$$

where *σ* = 0, if *A*_*z*_ ≥ 0; otherwise, *σ* = 1. *λ* = 1, if *A*_*y*_ ≥ 0; otherwise, *λ* = −1.

#### 2.4.2. System Realization

The overall system consists of front-end sensing subsystem and back-end data conversion subsystem. The front-end subsystem is the accelerometer sensor with a microcontroller for data acquisition and tilting angle transformation in digital formats. The angle information is then passed to the back-end subsystem for signal conversion into analog voltage signals to be hooked up with the PSG devices. Digital information has to be converted into analog signals because the tilting information has to be integrated with the PSG devices through their AUX inputs which are available for most of the PSG devices. Depending on how the data can be passed from front-end to back-end, the system can be realized as a wired system or a wireless system.

#### 2.4.3. Wired Sensing System Implementation

**Figure 5:**
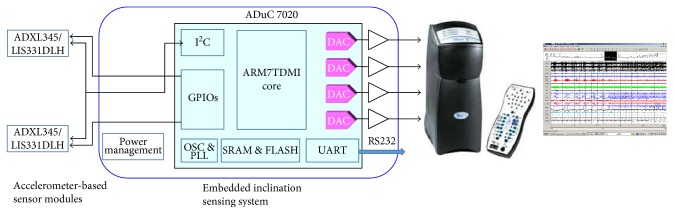
The system block diagram of a wired sensing system.

The wired system block diagram is shown in Figure 5 and mainly consisted of a microcontroller unit (MCU; ADuC7020; Analog Devices Inc.) and accelerometer modules (ADXL345, Analog Devices Inc.). The system was integrated into the PSG (Alice 5; Philips Respironics Inc.) for in-line and real-time application. The firmware executed on the MCU was developed in C language under the software of uVision4 IDE. The MCU core clock was set at 10.44 MHz. The accelerometer module was an ultralow-power high-performance 3-axis digital accelerometer. It has the sensitivity of 3.9 mg/LSB with a ±2*g* range and an on-chip low-pass filter for the removal of high-frequency noise. The communication between the accelerometers and MCU was through an I2C bus on a wired cable. The sampling rate of the accelerometer was set to be 100 Hz, and therefore, the MCU received the rectangular coordination (*A*_*x*_, *A*_*y*_, and  *A*_*z*_) of vector (−1*g*) from a 3-axis digital accelerometer for every 10 ms. The data were then transformed to the angular domain for tilting angle identifications. Four channels of on-chip digital-to-analog converters (DACs) were included in the MCU and converted the in-line calculated angles into analog voltages representing the inclination information. Then, the information was directly transferred to the auxiliary inputs of the PSG for the study of remission of the patient with OSAS. This is the system that has been implemented for the IRB tests.

#### 2.4.4. Wireless Sensing System Implementation

**Figure 6:**
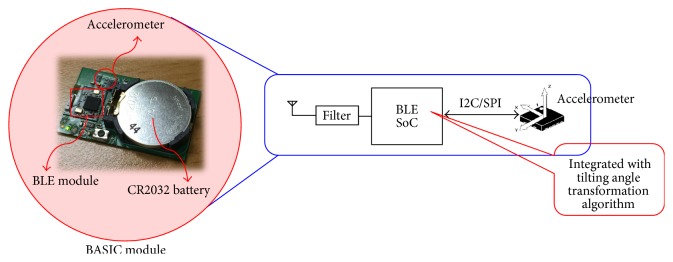
BASIC module and its block diagram.

If the tilting information of the head is transmitted wirelessly to the back-end data conversion subsystem and hooked up to the PSG, then it is the wireless sensing system. With this implementation, a front-end sensing subsystem could be attached on the forehead of the subject and without any cable connected to the PSG. In this front-end sensing subsystem, a module named “*BASIC*” was developed. BASIC stands for BLE-enabled Accelerometer-based Sensing In a Chip-packaging and is a fusion of A, B, and C technologies which are the accelerometer-based inertial sensing, Bluetooth low-energy (BLE) technology, and CORDIC-based tilting angle transformation algorithm. It is an integration of the accelerometer with the BLE module with CORDIC-based tilting angle transformation algorithm realized in the SoC of the BLE module. So, the module is capable of transmitting the tilting information according to the configured orientation over the air wirelessly by itself. The photo of the BASIC module and its block diagram is shown in Figure 6. With this module attached to the forehead of the subject, there will be no wired connection from the sensing front-end to the data conversion back-end which is then attached to the PSG device. The back-end system can be simply modified from Figure 5 using a BLE module to receive the transmitted data from the wireless sensing front-end subsystem, for example, the BASIC module; then, the received data are filled into the DACs inside the MCU for signal conversion and hooked up to the PSG device.

## 3. Results

In this section, the accuracy of the CORDIC-based tilting sensing algorithm-converted information is analyzed by comparing the angle calculated using mathematical equations as in (3). Then, the accuracy of the proposed sensing system was tested with a three-axis rotation platform to measure its actual accuracy under a precise controlled environment. Finally, the results of the system applied in a clinical IRB test is shown to prove the usability of the system for an unmet medical need.

### 3.1. Precision of the CORDIC-Based Tilting Sensing Algorithm

**Figure 7:**
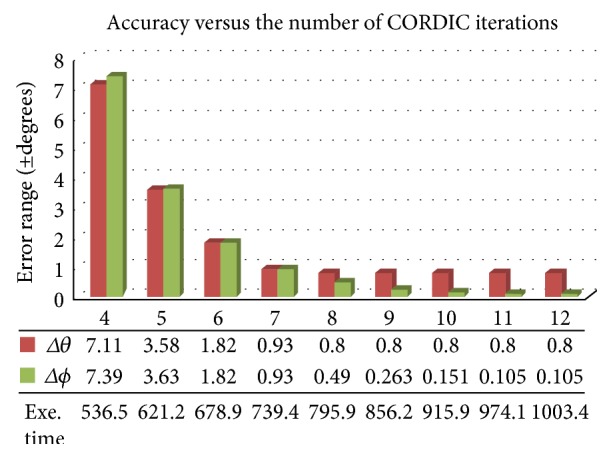
Accuracy and timing cost evaluation with varied number of iterations in CORDIC operations.

Validation of the algorithm accuracy was performed to show its precision. The number of iterations executed in the CORDIC-based algorithm can greatly impact the accuracy. Generally, the more iterations executed, the better accuracy can be obtained. But, the trade-off is to increase the computation time. An optimization was performed to find the number of iteration with acceptable accuracy and computation time. Hereby, different numbers of iterations from 4 to 12 were tested and compared with those of the calculated angles from the offline mathematical calculations. The results are summarized in Figure 7. It indicated that to meet the medical requirement of accuracy at 1°~2°, an acceptable accuracy of ±0.8 degrees for rotation angles and ±0.49 degrees for inclination angle was calculated by 8 iterations with the computation time of 795.9 ms. Such computation time was still much faster than the one from the original trigonometric calculation.

### 3.2. Accuracy of the Sensing System with an Accelerometer

**Figure 8:**
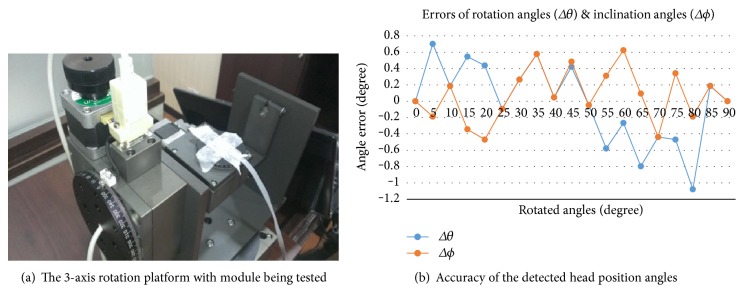
(a) The 3-axis rotation platform. (b) Errors of rotation angles and inclination angles of the proposed system.

The accuracy of the sensing system with the actual accelerometer was confirmed through testing on a controllable three-axis rotation platform (with a tri-axis step motor controller, TL-3T, from Tanlian E-O Co. Ltd.), as shown in Figure 8(a). The accelerometer sensing module being tested was placed at the center of the platform. During such testing, the tilting angles of the platform are controlled using precise step motors on the *x*-axis, *y*-axis, and *z*-axis with a resolution of 0.0025 degrees per step. In addition, the tilting angle of the platform is controlled incrementally and thoroughly. The calculated results from the proposed algorithm were then compared with the tilting setting of the platform. The accuracy analysis over the 0°~90° range is shown in Figure 8(b). The validation results show that the maximum error of the tilting angles transformed by the algorithm was within 1.08 degrees for the rotation angle and 0.6 degrees for the inclination angle. This kind of accuracy actually still can meet with the physician's requirement which is 1°~2° of accuracy for the study.

### 3.3. System Applied in the Institutional Review Board (IRB) Tests

**Figure 9:**
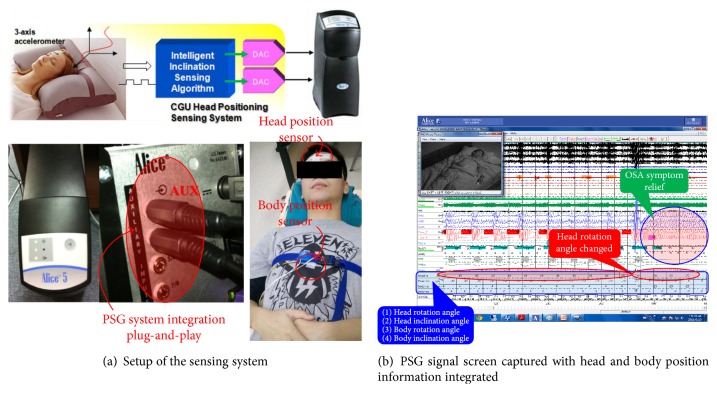
PSG screen captured with head and body position angle information integrated.

This study was reviewed and approved by the Institutional Review Board (IRB) of the Taipei Veterans General Hospital with 130 subjects participated. In the test, two sensors with the proposed system were attached to the subject's forehead for head position monitoring and the other one was attached to the subject's chest for body position monitoring as shown in Figure 9(a).  Figure 9(b) shows the PSG screen captured with the head and body position angle information integrated. This captured screen also catches the scenario that the patient had the head rotation angle changed just before remission from OSAS happened.

**Figure 10:**
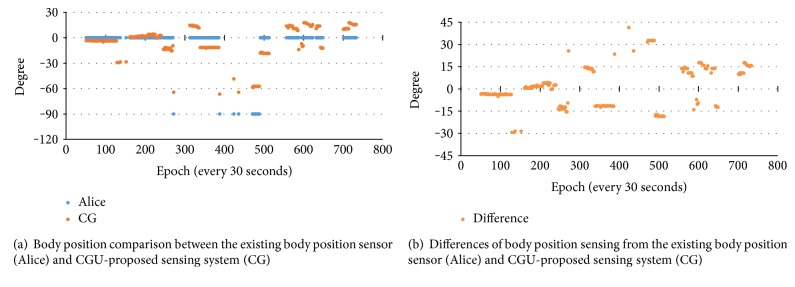
Body position comparison of the existing body position sensor (Alice) and CGU-proposed sensing system (CG).

As indicated above, one sensor with the proposed sensing system was also attached to the chest for body position monitoring. Since the PSG system was also hooked up with its existing body position sensor which can only provide very rough resolutions classified by 45 degrees (i.e., 0°, −45°, −90°, −135°, −80°, −225°, −270°, and −315° ranges) into 8 sleep positions, a comparison of the body position from these two sensors is provided in Figure 10, where Figure 10(a) shows the details of the body position angle from two sensors, the existing body position sensor (Alice), and the CGU-proposed sensing system (CG) in this study, and Figure 10(b) shows the differences from these two. In this comparison, the epoch in the *x*-axis of both Figures 10(a) and 10(b) represents a 30-second time period which is a standard unit of time used in clinical sleep study. Data were from the PSG of one patient's overnight sleep test. In Alice (the PSG used in this study), the sampling rate for body position sensor is 1 Hz, and the CGU-proposed sensing system in this study could achieve the sampling rate of 100 Hz. Hence, the 1st data point of every one second from the proposed sensing system was used to compare with the data from existing body position sensors. The position angles shown in Figure 10 were recorded whenever there was apnea/hypopnea that occurred; therefore, data points in Figure 10 were not continuous. It can be observed that the sensing system proposed in this study delivered higher resolution of the position angles comparing with the existing body position sensor of the PSG system which had been used by other researchers [4, 5] for the study of head position-related issues on OSAS patients when a higher resolution of head position-sensing system was not yet available for clinical diagnosis.

Table 1 shows the statistics information of the body position comparison shown in Figure 10. It can be observed that the body position angle differences between the existing body position sensor and the CGU sensing system described in this study are ranging from −29.40° to 41.38° with the average of the absolute differences at 10.50° and a standard deviation of 8.02°. Even by subtracting the maximum error, 1.08°, in the rotation angle, of the CGU sensing system found in Section 3.2 validated with a commercially available controllable rotation platform, the differences of the angles reported by the existing body position sensor are still pretty significant compared with the actual angles. It can be concluded that using the existing body position sensor is not accurate enough to deliver the head position information due to its low resolution. In fact, the CGU sensing system proposed in this study can deliver more accurate position (orientation) information of the desired portion of the body, including the head. Hence, it is more suitable for the remission study of OSAS patients correlated with the head position.

## 4. Discussion

### 4.1. Limitations of Using Accelerometer-Based Inclination Sensing for Head Positioning

There are also some limitations using accelerometer-based inclination sensing for head positioning:

- The accuracy of accelerometer-based inclination sensing basing upon the assumption of measured net force is gravitational acceleration only. If the object attached to the accelerometer has significant continuous movements, then the measured acceleration vector from the accelerometer will be the combination of the movement force with the gravitational acceleration. Fortunately, the head movements during sleeping are typically occasional and gentle. Therefore, once the head movements stop, the accurate head position information could be retrieved through accelerometer-based inclination sensing.
- If in case the measured gravity vector in accelerometer is almost aligned to one of the axes and if the subject rotates along that axis, then the accelerometer is not able to differentiate the rotation angle. This situation happens when the subject sits up or even stands up, such that the gravity vector in the accelerometer is aligned to the *x*-axis, as shown in Figure 1(a). Under this circumstance, the projected gravity vector on the *y*-*z* plane is 0 (*A*_*y*_ = *A*_*z*_ = 0). As a result, it is not able to calculate the rotation angle *θ* as in (3). However, the system is supposed to detect head position when the subject is sleeping, and when the situation happens, the subject could not be sleeping. Therefore, this is the case of no interest for sleeping diagnosis.
- When the subject leans forward, the head position will be like the figure shown in Figure 1(b). In this case, the *A*_*z*_ reading from the accelerometer will be negative and *A*_*y*_ ≒ 0. As described in (3), to extend the rotation angle *θ* to cover the whole 360° range, the term of *σ* · *λ* · 180 is used for the adjustment. Since *A*_*z*_ < 0, and *A*_*y*_ ≒ 0, so, even with small changes of *A*_*y*_, it results in the sign changes of *A*_*y*_. Consequently, the calculated rotation angle *θ* will be jumping between +180° and −180°. This phenomenon is also observed in our study. However, this is a case of no interest since this situation happens when the subject sits up and leans forward. Under this circumstance, the subject is not in a normal sleeping mode.

### 4.2. Error Ingredient of the Proposed Sensing System

In the accuracy validation of the proposed sensing system, the errors of rotation angles and inclination angles are actually larger than the errors shown in the precision analysis of the CORDIC-based tilting sensing algorithm alone. This is because the overall errors of the sensing system also include the errors from the accelerometer itself besides the errors contributed by the algorithm. Even though the data from the accelerometer were calibrated, the data from the accelerometer still contains some noise as well as the mechanical structure of the accelerometer. During the system validation, it can be observed that the 3 axes of the accelerometer were not perpendicular to each other. This would add to the overall errors of the proposed sensing system using accelerometers.

## 5. Conclusions

This study presents the design and development of a sensing system for head position monitoring. The system has been realized and was proved to be able to plug-and-play into most of the PSG devices used in hospitals and clinical sleep institutions for the diagnosis of OSAS patients. A CORDIC-based tilting sensing algorithm was also implemented in the system to reduce the burdens of high computability required, such that the cost of the system can be reduced. The system has been carefully verified with the performance analysis of the tilting sensing algorithm and accuracy validation through a precise controlled rotation platform. The system was proved to meet the medical needs of doctors on providing accurate head position information for their study of remission in OSAS patients.

## Figures and Tables

**Figure 11:**
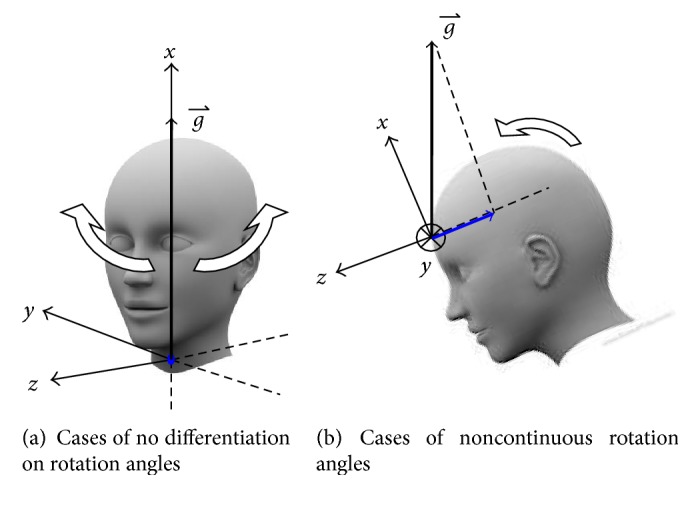
Special cases for accelerometer-based inclination sensing of head positioning monitoring.

**Table 1:** Statistics information of body position comparison for Figure 10.

Statistics of angle difference	Value
Average of absolute differences	10.50°
Standard deviation of absolute differences	8.02°
Maximum difference	41.38°
Minimum difference	−29.40°
